# Predicting the effects of frameshifting indels

**DOI:** 10.1186/gb-2012-13-2-r9

**Published:** 2012-02-09

**Authors:** Jing Hu, Pauline C Ng

**Affiliations:** 1 Department of Mathematics and Computer Science, Franklin and Marshall College, 415 Harrisburg Ave, Lancaster, PA 17603, USA; 2 Computational and Mathematical Biology, Genome Institute of Singapore, 60 Biopolis St, Singapore, Singapore 138672

## Abstract

Each human has approximately 50 to 280 frameshifting indels, yet their implications are unknown. We created SIFT Indel, a prediction method for frameshifting indels that has 84% accuracy. The percentage of human frameshifting indels predicted to be gene-damaging is negatively correlated with allele frequency. We also show that although the first frameshifting indel in a gene causes loss of function, there is a tendency for the second frameshifting indel to compensate and restore protein function. SIFT Indel is available at http://sift-dna.org/www/SIFT_indels2.html

## Background

Small insertions/deletions (indels of 20 bp or less) account for nearly 24% of known Mendelian disease mutations. It is the second largest class of mutation type that leads to disease following amino acid substitutions, which account for over half of known Mendelian disease mutations [1]. There exist many bioinformatics algorithms that predict whether an amino acid substitution affects protein function (for example, SIFT [2], PolyPhen [3]; see [4] for a review), and these are commonly used for predicting and prioritizing disease variants, but very little work has been done for indels [5]. Because indels account for a significant fraction of known disease-causing mutations, an algorithm that can clearly distinguish between neutral and gene-damaging indels would be useful.

Historically, indels have been less studied compared to single nucleotide variants and structural variation. Indel identification is challenging for Sanger and next-generation sequencing, although advances have been made [6-9]. Mills *et al*. [10] identified 1.96 million indels from Sanger reads in the NCBI trace archive that showed relatively low overlap with dbSNP, 1000 Genomes, and five personal genomes. This indicates that indel discovery has not reached saturation. As more indels are identified, the challenge will be to characterize these new variants.

Indels in coding regions of the genome that have lengths that are not divisible by three may cause frameshifts. The mutant mRNA may be subsequently degraded by nonsense-mediated or non-stop-mediated mRNA decay [11-13]. Researchers tend to assume these frameshifting (FS) indels are loss-of-function variants. However, we and other researchers have identified some trends for FS indel variants observed in the human population. For example, polymorphic indels tend to cluster towards the end of a protein, thereby avoiding nonsense-mediated decay [14,15]. They also tend to occur in hypothetical and olfactory genes, which are under relaxed selection [14]. This suggests that some FS indels could be functionally neutral.

Each individual human genome can contain approximately 50 to 280 small FS indels [16,17]. However, identification of FS indels is prone to sequencing, mapping, and annotation errors so the real number is likely to be towards the lower end of this range [17]. With inexpensive and ubiquitous genome sequencing, it would be time-consuming to analyze these hundreds of mutations manually, yet it would be important to distinguish the functionally neutral indels from those that are under negative selection. We present the SIFT indel algorithm, which predicts the effects of indels at 84% accuracy. This is an extension to the SIFT algorithm, which predicts the effect of amino acid substitutions [2,18-20]. We show that the percentage of FS indels predicted to be gene-damaging is negatively correlated with allele frequency. We also show that genes with FS indels are dynamically evolving between nonfunctional and functional forms.

## Results

### Classifier construction and performance

We construct a classifier based on the decision tree algorithm to predict if an indel is 'gene-damaging' (affects the function of the gene it resides in) or 'neutral' (does not affect gene function). The SIFT Indel classifier was trained to distinguish between two datasets: (1) a set of disease-causing FS indels, and (2) functionally neutral indels. The disease-causing FS indels were taken from the Human Gene Mutation Database (HGMD) [21], a database of disease mutations found in patients. The neutral indels consisted of coding indels with sizes not divisible by three that were derived from pairwise alignments of human with cow, dog, horse, chimpanzee, rhesus macaque, and rat [22] (Materials and methods).

Decision tree algorithms have been widely applied to many bioinformatics problems, including the classification of SNPs [23-25]. One of the benefits of decision tree algorithms compared with other black-box machine learning algorithms (for example, neural networks, support vector machine, and so on) is that it provides interpretable classification rules, which might provide insight about the mechanism behind the classification. We therefore constructed a decision tree to distinguish between the gene-damaging and neutral indels. Disease-causing indels are treated as the positive class, while neutral indels are treated as the negative class. Sensitivity is the fraction of disease-causing indels that are correctly predicted as gene-damaging. Specificity is the fraction of neutral indels that are correctly predicted as neutral. Precision is the percentage of predicted gene-damaging indels that are actually gene-damaging. Accuracy is the percentage of overall predictions that are correct.

For each indel, 20 features are extracted describing the indel and its influences on the gene product (Table S1 in Additional file 1) [11-13,26-30]. When all 20 features are used, the decision tree achieved an average performance of 85% sensitivity, 81% specificity, 82% precision, and 83% accuracy across ten experiments (Table 1). Because the number of disease indels and neutral indels (1,292 versus 2,602) is not balanced in our non-redundant dataset, in order to avoid training bias, we used all 1,292 disease indels and randomly sampled 1,292 neutral indels from the neutral dataset for training and cross-validation. To ensure that the sampling process does not significantly affect the prediction performance, we conducted ten ten-fold cross-validation experiments. For each experiment we resampled the neutral indel dataset, and a ten-fold cross-validation process was used to evaluate the classification performance. The standard deviations were within very reasonable range (that is, 1.3%, 1.2%, 0.9%, and 0.8%, respectively), which shows that sampling does not have much influence on the prediction performance and it is safe to use sampling to train the final decision tree.

**Table 1:** Performance of the decision tree using different features

Features used	Sensitivity ± SD	Specificity ± SD	Precision ± SD	Accuracy ± SD
20 features	85 ± 1.3%	81 ± 1.2%	81 ± 0.9%	83 ± 0.8%
4 selected features	90%	78%	81%	84%

SD, standard deviation.

**Figure 1:**
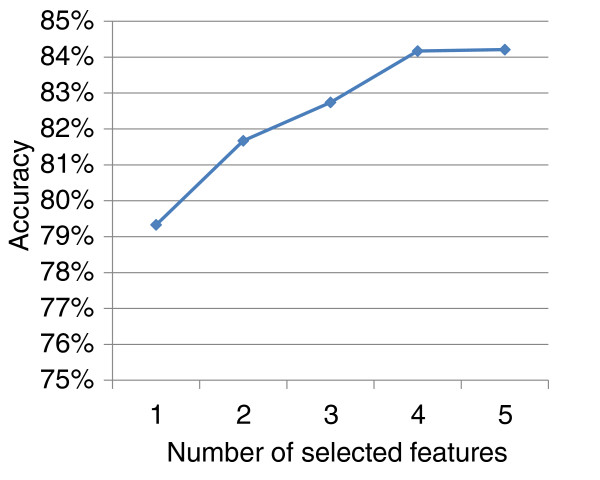
**The classification performance of the decision tree is improved as the feature selection progresses until the number of selected features reaches four, where the decision tree reaches its best accuracy evaluated by using ten-fold cross-validation**. The prediction accuracy does not change significantly when five features are selected.

However, not all the 20 features are equally useful for the prediction of FS indels. Also, some features might be correlated with each other, which can impair prediction performance. We therefore used one of the samplings and applied a greedy feature selection method to select the most relevant features by adding one feature at a time. In each iteration for the feature selection process, the feature that showed the largest improvement in performance was chosen. As can been see from Figure 1, the decision tree reaches its maximum performance in terms of classification accuracy after four features are chosen. The four selected features in the order of being chosen are: 1) fraction of affected conserved DNA bases; 2) indel location relative to the transcript, and taking the maximum across all transcripts; 3) fraction of affected conserved amino acids, taking the maximum across all transcripts; and 4) minimum distance of indel to the exon boundary of all affected transcripts. They are features 14, 5, 18, and 15, respectively, in Table S1 in Additional file 1 where more detailed descriptions can be found. The final method uses these four features and achieves 90% sensitivity, 78% specificity, 81% precision and 84% overall accuracy (Table 1). The final method has better performance than using all twenty features.

When calculating conservation, there is a possibility for circularity because DNA and protein conservation scores use the same mammalian sequences that we used to construct our neutral indel data set. We disprove that circularity is an issue. For DNA conservation, we used PhyloP scores and PhyloP treats gap positions as missing data [29]. Therefore, there is no circularity when using DNA conservation. Protein conservation scores were calculated as described in Table S1 in Additional file 1 where a protein multiple sequence alignment was constructed from vertebrate sequences and conservation scores derived from the alignment. To check that circularity is not a factor, we reconstructed the multiple sequence alignment without the sequences from which neutral indels were derived and recalculated conservation scores. Performance was not significantly affected when these conservation scores were used (accuracy 84% versus 83%; Table S2 in Additional file 1).

### Contribution of selection features and classification rules

One of the benefits of a decision tree is that it provides us with classification rules, which can provide some biological insights. For a decision tree, the tree's internal nodes test features while the tree's leaves make decisions. A classification rule and its corresponding thresholds are automatically extracted by following the decision path from the root of the tree to one of its leaves. This is a non-heuristic process. The confidence score is the fraction of training samples that are correctly classified using a given path. From the trained decision tree, there are twelve classification rules derived (see table in [31]), among which four not only cover most of the training samples but also have high confidence scores. These rules are as follows.

Rule 1: if the percentage of affected conserved DNA bases is very small (< 1.2% of all conserved DNA bases of the gene), then the indel will not affect gene function. The confidence score for this rule is 0.96. (There were 687 data points that followed this rule; 660 were correctly classified as neutral.)

Rule 2: even if the maximum relative indel location is not near the end of the coding sequence (≤ 85.5%), then the indel is still neutral as long as the percentage of affected conserved DNA bases is relatively low (≤ 4.3%). This explains why there are some indels in the middle of cDNA sequence (which could be nonconserved alternatively spliced exons), but are still functionally neutral. The confidence score for this rule is 0.92. (There were 129 data points that followed this rule; 118 were correctly classified as neutral.)

Rule 3: if the percentage of affected conserved DNA bases is relatively low (≤ 4.3%), the maximum fraction of lost conserved amino acids is very low (≤ 0.9%), and maximum indel position is near the end of the cDNA sequence (> 85.5%), then the indel is predicted to be functionally neutral. The confidence score for this rule is 0.81. (There were 102 data points that followed this rule; 83 were correctly classified as neutral.)

Rule 4: if more than 6.2% of the conserved DNA bases are affected, conserved amino acids have been lost (> 0.9%), the indel position is in the middle of one of the cDNA sequences (maximum relative indel location > 8.7%), and the indel is also in the middle of the exon (minimum distance of indel to the exon boundary is > 6), then the indel is likely to be gene-damaging. The confidence score for this rule is 0.86. (There were 1,193 data points that followed this rule; 1,024 were correctly classified as gene-damaging.)

From the training dataset, 66.6% (861) neutral indels follow rules 1 to 3 and 79.3% (1,024) gene-damaging indels follow rule 4. Therefore, these four rules represent the majority of training samples. Together, these rules reflect the biological knowledge that if an indel affects a very small percentage of conserved DNA bases and causes a very small fraction of conserved amino acids to be lost in the resulting protein, then very likely the indel will have no significant effect on gene function. An indel is more likely to be gene-damaging if the indel affects a high percentage of conserved DNA regions and/or amino acids, and the indel tends to be in the middle of a cDNA sequence and exon.

One concern with our neutral dataset is that it may contain indels arising from sequencing errors [32]. Therefore, as further validation to our algorithm, we applied the final four-feature algorithm to additional neutral datasets. In our first set, we examined indels observed in at least two species and with no other gaps within 30 bp (*n *= 167). Requiring the indel to be observed independently in at least two species reduces erroneous indel calls. The 30-bp threshold was based on the observation that neighboring non-3n indels can compensate for a frameshifting non-3n indel, thus restoring gene function (see latter section 'Fixed loss-of-function indels in other mammalian genomes'). In this small but highly filtered neutral dataset, high specificity 87% (145/167) was observed. This high-quality neutral dataset minimizes indel call errors but its small size (*n *= 167) prevents us from using it as a training set because this would lead to over-fitting of the decision tree [33]. When we lowered the 30-bp threshold to 5 bp, the data set increased in size but specificity dropped to 63% (1,961/2,960). However, manual inspection of 20 indels incorrectly predicted as gene-damaging from this dataset showed that 40% (8/20) had nearby compensatory non-3n indels. After correcting for this, the estimated specificity is approximately 77%, which is close to the final method (78%).

### Human indels

We applied the SIFT Indel algorithm to the FS indels identified from the human genomes sequenced by the 1000 Genomes Project (1000G) [34] and by Complete Genomics, Inc. (CGI) [35]. The 1000G has identified indels from low-coverage genome sequencing of Europeans, Asians, and Africans. CGI has sequenced a diversity panel that contains a smaller number of individuals, but at higher depth (69 individuals from 11 different populations). The allele frequencies of 1000G indels are population-specific, while the allele frequencies for CGI indels are global because they are based on the diversity panel. Analyzing both 1000G and CGI datasets permits analysis at global (CGI) and population-specific (1000G) levels.

**Figure 2:**
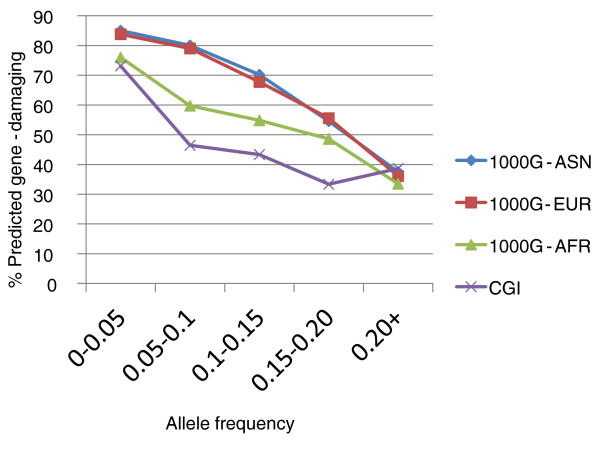
**Allele frequency distribution for frameshifting indels predicted to affect protein function in Asian (ASN), European (EUR), and African (AFR) populations from the 1000 Genomes Project (1000G) low-coverage sequencing project and Complete Genomics, Inc. diversity panel (CGI)**.

The majority of FS indels were predicted to be gene-damaging for both the 1000G and CGI datasets. In the 1000G dataset, 79% (2,259/2,852) were predicted to be gene-damaging in Europeans, 80% (2,683/3,332) in Asians, and 70% (1,585/2,278) in Africans. In the CGI dataset, 73% (973/1,334) of indels were predicted gene-damaging. This high percentage can be explained by the fact that most FS indels are rare. When binned by allele frequency, the percentage of FS indels predicted to be deleterious is negatively correlated with allele frequency (Figure 2), which is the trend that has been previously observed for nonsynonymous mutations [36]. However, the trend is much more severe for FS indels. For rare indels (allele frequency < 0.05), approximately 80% are predicted to affect function (Figure 2), compared to 20 to 40% for nonsynonymous variants that was previously reported [36]. For common FS indels (allele frequencies > 0.20), 33 to 39% are predicted deleterious in the different datasets, whereas less than 5% of nonsynonymous variants are deleterious [36]. This is consistent with the observation that indels are under stronger purifying selection than nonsynonymous variants [10]. Even for common indels with allele frequencies between 0.10 and 0.20, a substantial proportion are predicted gene-damaging, approximately 65% for the Asian and European populations, 53% for Africans, and 40% for the CGI dataset. Geneticists typically use an allele frequency cutoff of 0.05 for neutral SNPs but these results indicate that a significant number of common FS indels are gene-damaging and an allele frequency threshold of 0.05 for FS indels may be too low.

We questioned why there is a high proportion of gene-damaging indels that have relatively high frequencies in the human population. There are several possible explanations for this. The first explanation is that common gene-damaging indels are in genes under relaxed selection [14,17]. For example, genes with common gene-damaging FS indels in humans (frequency > 0.10) are overrepresented in the sensory perception of smell by two-fold (*P *= 1.23 × 10^-5^). This is not surprising since olfactory receptors are under relaxed selection in humans [37].

The second explanation is that a higher proportion of deleterious alleles accumulate in a bottlenecked population [38]. When a population undergoes a bottleneck and then expansion, deleterious variants can become common because there is not enough time for purifying selection. The European and Asian populations have undergone bottlenecks [38,39], and as can be seen in Figure 2, these two populations have a higher percentage of predicted gene-damaging FS indels compared to the African population and the CGI diversity panel.

A third possible explanation for common gene-damaging indels is positive or balancing selection for the variant. Some of the genes with common gene-damaging indels have supportive evidence for undergoing positive selection. We found common gene-damaging indels in the CYP3A gene cluster. The CYP3A cluster has been shown to be under positive selection [40,41], and mutations are favored according to the 'sodium retention hypothesis' [42], which proposes that human populations living in hot, humid areas preferentially retain salt. Our global analysis detected the CYP3A5*7 allele [43] as a common damaging 1-bp insertion in the *CYP3A5 *gene; this gene is involved in sodium transport and has been proposed to play a role in hypertension [41,44]. The gene-damaging indel allele CYP3A43*2A/B was also detected in the analysis. Finally, there is a common gene-damaging indel in *HERC2*, a gene that has been found to be associated with blue eye color, and the association follows a north-south gradient distribution across the European populations [45].

### Fixed loss-of-function indels in other mammalian genomes

In 1999, Maynard Olsen proposed the 'less is more' hypothesis, where gene loss (which can result from FS indels) is advantageous for species' survival [46]. For example, a 32-bp deletion in the gene *CCR5 *causes 'less' gene function, yet protects against HIV ('more' fitness) [47]. We concentrated on inter-species variation to explore this hypothesis. We examined the genes with fixed indels in the other mammalian genomes. These indels were part of our neutral training dataset, where we had assumed FS indels in mammalian genomes were functionally neutral. However, it is possible that some of these indels do affect gene function, and have been advantageously fixed due to positive selection. There were 679 genes in the mammalian species that contained FS indels predicted to affect gene function. We looked at these 679 genes to see if they share the characteristics of genes under positive selection. It has been previously shown that genes under positive selection in mammals have functions such as defense/immunity, chemosensory perception, and extracellular space [48,49]. We found that the number of genes with predicted gene-damaging FS indels was enriched in defense function 1.76-fold (*P *= 0.012) and in extracellular space by 1.36-fold (*P *= 0.0012) according to Gene Ontology [50]. Interestingly, olfactory genes were underrepresented almost four-fold (*P *= 0.005), and this may be because the indels are derived from species where the sense of smell is important (for example, rat and dog).

**Figure 3:**
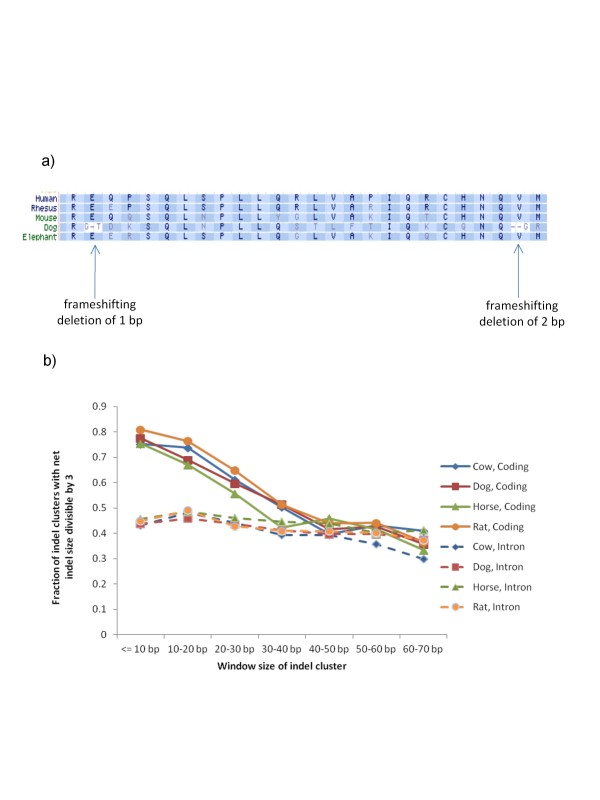
**Frameshifting coding indels close together tend to compensate for each other**. **(a)**An example of compensatory FS indels. The dog orthologue of human FLJ43860 contains two FS indels. Individually, they cause frameshifts, but together in-frame translation is restored. **(b) **Multiple FS indels within a window size were clustered, and their net change in size was calculated. On the y-axis is the fraction of clusters with indel net size divisible by three, where amino acids between the two FS indels would change, but ultimately in-frame translation is restored. Solid lines are used for multiple FS indels in coding exons; dashed lines are for intron regions.

According to Olson's 'less is more' hypothesis [46], 'once <*a gene's >*function is lost - unless the lesion involves a complete deletion of the gene - the mutated gene will persist in the genome and may be available for reversion if the selective environment shifts once more.' If a FS indel happens in a gene, the gene's function can be restored if there is a second compensatory indel that restores in-frame translation. In this scenario, while both indels' sizes are not divisible by three, the net size of the two indels together is divisible by three. An example is shown in Figure 3a; when the human and dog protein sequences for FLJ43860 are aligned with each other, the dog genome has a 1-bp deletion in the gene, but 67 bp downstream of the deletion, an additional 2 bp are also deleted, so that in-frame translation is restored. While it is unknown which event occurred first (the 1-bp deletion or the 2-bp deletion), the first event had to render the gene functionless, only to be rescued by the second.

We provide two pieces of evidence to show that such compensatory events occur more frequently than expected in mammalian species. We analyzed the full set of FS indels observed in mammalian genomes when aligned to human. We demonstrate that FS indels near each other are more likely to restore the translation frame. We look at windows with at least two indels with a certain distance from each other on the transcript. We calculate the net size of FS indels in the window. If the indels are within 20 bp of each other, there is more than 70% chance that the multiple FS indels together have a net length size that is divisible by three and the translation frame is restored (Figure 3b). In comparison, the same analysis on 10,000 intronic regions shows that only 45% of indel clusters are divisible by three. The intronic regions serve as a control for possible sequencing and alignment artifacts. As the indels become more distant, the restoration effect diminishes around 40 to 50 bp (approximately 15 amino acids). It is logical that a compensatory FS indel would be preferentially located near the first FS indel in order to minimize changes in protein sequence. The second piece of evidence that supports compensation is that when two indels occur in the same exon, their net size is divisible by three more often than expected by chance. We looked at exons that contained two FS indels in mammalian genomes when aligned to humans, and calculated the net size of the FS indel pair. As a control, we performed the same calculation for introns (200-bp regions). When two FS indels occur together in an exon, it is 1.3- to 1.9-fold more likely that the net size of the two indels will be divisible by three compared to introns containing two indels (Table 2). This supports further evidence that there is selection for compensation.

**Table 2:** Observed fractions for the net size of two nearby indels

	Fraction of two indels in 200-bp intron that have net size divisible by 3 (control)	Fraction of two indels in the same exon that have net size divisible by 3	Enrichment (exon fraction/intron fraction)
Cow	0.45	0.77	1.71
Dog	0.47	0.78	1.64
Horse	0.49	0.66	1.33
Rat	0.46	0.87	1.92

All enrichment ratios are statistically significant (*P *< 0.001) by Fisher's exact test.

## Conclusions

We present here the SIFT Indel prediction algorithm for FS indels that provides good separation between neutral and gene-damaging with 90% sensitivity, 78% specificity, 81% precision and 84% overall accuracy. The accuracy of a prediction algorithm is highly dependent on the training datasets. For this algorithm, we trained on indels found in patients and interspecies indels. Similar training datasets were used by a popular amino acid substitution prediction algorithm PolyPhen [3,51]. Prediction algorithms have also used human polymorphic variation as training datasets, and this can be used in the future if the numbers are sufficiently large. If human polymorphic indels are used for training prediction algorithms, our results in Figure 2 indicate that it is best to use indels from African or diverse genomes because there are some common deleterious indels in bottlenecked populations such as the Europeans and Asians.

Functionally neutral indels may be due to location or gene annotation errors, or because the indels themselves reside in pseudogenes or indispensable genes [14,17]. The four features in our final algorithm most likely capture location and gene annotation errors. For example, the feature of 'minimum distance to exon boundary' may capture gene annotation errors for indels near splice junctions that have not been correctly annotated. Despite including gene-specific features, our final algorithm did not incorporate any gene-specific features such as K_a_/K_s _that typically mark pseudogenes or indispensable genes. This is likely due to our neutral indel training dataset, where we purposely excluded genes with more than one FS indel; thus, pseudogenes, indispensable, and quickly evolving genes were not part of the training dataset. Existing algorithms that rank gene importance [52,53] could be used in combination with our method to prioritize gene-damaging indels that also have phenotypic consequence.

A high proportion of FS indels observed in humans are predicted gene-damaging, but most of these FS indels are rare. As expected, the percentage of deleterious indels is negatively correlated with allele frequency. Geneticists often use allele frequency to analyze genetic variation, and it is often presumed that common variants are neutral (for example, SNPs with minor allele frequency > 0.05 considered neutral). Our results show that a significant proportion of common indels are likely to have an impact, especially in the European and Asian populations that have undergone a bottleneck. Hence, our study suggests that filtering out common FS indels by allele frequency alone may lead to missed phenotypic variation. SIFT Indel, in conjunction with allele frequency and gene function, provides additional support whether or not to filter out the indel. We suggest the following criteria for FS indels. If the indel is found in multiple populations and predicted to be neutral by the prediction algorithm, then it should be given a lower priority. Common indels should be considered high priority if found in only one population, located in functionally relevant genes, and predicted gene-damaging. This rule holds especially true for variants detected in bottlenecked populations.

**Figure 4:**
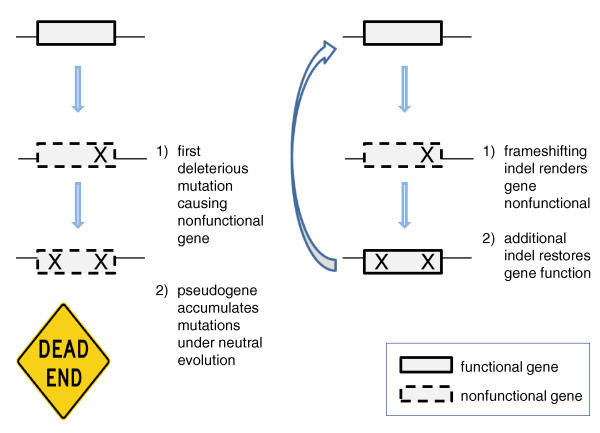
**The 'less is more' hypothesis by Maynard Olson proposes that compensatory mutations can restore gene function**. In the classical thinking of pseudogene evolution, once a gene has a deleterious mutation that becomes fixed, more mutations will accumulate because there is no longer any selective pressure to retain gene function. In the 'less is more' model, while the first frameshifting indel causes loss of function, it is possible for a subsequent frameshifting indel to restore gene function if it restores in-frame translation.

We also show that a FS indel is not an evolutionary dead end, but a gene with a FS mutation may eventually revert back to a functional gene (Figure 4). Specifically, we show that gene reversion with a second compensatory FS indel is observed more often than expected by chance. Compensation is much easier for coding indels than for coding single nucleotide variants. Coding single nucleotide mutations that cause amino acid substitutions may not completely knock out gene function, and it would be difficult to revert back to normal function because either that same exact nucleotide has to mutate back or a compensatory mutation at the amino acid level has to occur. For single nucleotide changes that introduce pre-termination stop codons, the mutation space is more limited because only certain codons can mutate to a stop codon, and reversion will only occur if that same exact nucleotide mutates back. In contrast, a coding indel that occurs almost anywhere in the gene will knock out gene function (with the exception of the rules identified in our SIFT Indel classifier). In order to regain function, a nearby compensatory FS indel can suffice. For example, a 1-bp deletion can be rescued by another 1-bp insertion or a 2-bp deletion, and the second indel does not have to be at the same exact location. Thus, indels are far more flexible than single nucleotide variants in creating loss of function, and subsequently rescuing itself. This would be desirable in changing environments.

## Materials and methods

### Datasets

The SIFT Indel classifier was trained on two datasets: (1) a set of disease-causing FS indels, and (2) functionally neutral indels.

#### Indel disease set

In this study, indels found in the disease genes of affected patients were assumed to be gene-damaging and used for training and testing purposes. This disease-causing set was obtained from HGMD version 2010.2 [21]. HGMD is a database of disease mutations found in patients and it provides chromosomal coordinates for each indel. There were 20,107 FS indels in this dataset from 1,373 genes. We chose one indel per gene to avoid over-training on certain genes. After removal of indels from non-exon regions and from genes with invalid/incomplete transcripts, there were 1,292 disease indels in the final dataset used for the development of the algorithm.

#### Neutral indel set

Indels with sizes not divisible by three were derived from pairwise alignments from the UCSC genome browser of human with cow, dog, horse, chimpanzee, rhesus macaque and rat [22] (designated as bosTau4, canFam2, equCab2, panTro2, rheMac2, and rn4, respectively). The assembled genomes of these organisms were syntenically aligned with human. Mouse/human alignments were not used because mouse did not have quality sequencing scores available. The multiple sequence alignment of these species (UCSC multiz) was not used for training because indel identification was confounded by regions that had many gaps. Only indels in high-quality sequences were kept: the 10-bp sequence surrounding the indel was required to have quality scores of 9. In order to prevent including indels from pseudogenes and misalignments, only one FS indel per gene was allowed. If more than one FS indel was observed, the gene was assumed to be a pseudogene and all indels from that gene for that organism were removed from the dataset. We combined all the indels from the different species together and randomly chose one indel per gene. After removal of indels from genes with invalid/incomplete transcripts, there were 2,602 neutral indels in the final dataset used for the development of the algorithm.

In addition to the neutral indel dataset used for training as described above, two other neutral indel datasets were constructed to assess algorithm performance. Indels not divisible by three were derived from the UCSC multiz alignment. To minimize erroneous indel calls, we kept indels that were identically observed in at least two species from the same lineage. For example, an indel event unique to mouse and rat (rodent lineage) would pass our filters, but an indel event observed in mouse and dog but not rat would be discarded. For the first indel dataset, any indel within 30 bp of another indel was discarded. This removed indels that were called due to misalignment or in regions evolving neutrally or quickly, and thus this dataset is composed of indels that we have high confidence in. Because this dataset was small (*n *= 167), we decreased the cutoff for neighboring indels from 30 bp to 5 bp to obtain a larger but lower quality dataset (*n *= 2,960).

We used Ensembl build 37, v. p3 (Ensembl Genes 63) for gene annotation [26]. Human indels from the 1000G were based on the 4 August 2010 release; February 2011 Data Update. Human indels from the CGI diversity panel were downloaded from [35].

### Prediction algorithm

We used the J48 decision tree algorithm implemented in WEKA [54]. Because the number of neutral indels is more than twice the number of disease indels in the final dataset, to avoid training bias toward neutral indels, we kept all the disease indels and randomly picked an equal number of neutral indels for training and testing of the algorithm.

### Performance measurement

Ten-fold cross-validation was used to evaluate the method. The dataset was divided into ten subsets. In each round of the experiment, nine subsets were used as the training set, and the remaining subset was used as the test set. This procedure was repeated ten times, with each subset being used as the test set once.

Performances are measured using sensitivity, specificity, precision, and accuracy, which are defined as:

sensitivity=TP/(TP+FN)

specificity=TN/(TN+FP)

precision=TP/(TP+FP)

accuracy=(TP+TN)/(TP+FN+TN+FP)

where TP is the number of true positives (that is, the number of disease-causing indels predicted as gene-damaging); TN is the number of true negatives (that is, the number of neutral indels predicted as neutral); FN is the number of false negatives (that is, the number of disease-causing indels predicted as neutral); and FP is the number false positives (that is, the number of neutral indels predicted as gene-damaging).

### Feature selection

There were 20 features extracted describing each indel and its influences on the gene product (Table S1 in Additional file 1). We applied a greedy feature selection method to select the most relevant features by adding one feature at a time. This feature selection method has been used previously [25]. Let *S *be the set of the selected features, *A *be the set of available features, and *N *be the size of *A*. Initially, *S *is empty and *N *= 20. Features were added into *S *incrementally using the following procedure:

1. Pick one feature *f *from *A*.

2. Build the decision tree using the union of feature *f *and all features in *S*, and then evaluate the classifier using ten-fold cross validation by optimizing for accuracy.

3. Repeat steps 1 and 2 *N *times, so that every feature in *A *is tested once. The feature that brings the biggest improvement in classification performance is removed from *A *and added into *S*. The size of *S *is increased by 1 whereas the value of *N *is decreased by 1.

To avoid over-fitting, the procedure continued until including more features into *S *does not increase the performance significantly (that is, accuracy improves less than 0.1%). In the end, four features were added to *S *and chosen.

## Abbreviations

1000G: 1000 Genomes Project; bp: base pair; CGI: Complete Genomics, Inc.; FS: frameshift; HGMD: Human Gene Mutation Database; indel: insertion/deletion; SNP: single nucleotide polymorphism.

## Competing interests

The authors declare that they have no competing interests.

## Authors' contributions

JH designed and implemented the decision tree algorithm. PN conceived of the idea and did the analysis for human and mammalian indels. Both authors drafted, read, and approved the final manuscript for publication.

## Supplementary Material

Additional file 1 **Supplemental tables and figures**. Click here for file
