# Supplementary material for: Predicting the effects of frameshifting indels
Source: Genome Biol. 2012 Feb 9;13(2):R9. doi: 10.1186/gb-2012-13-2-r9 (PMC3334572; doi:10.1186/gb-2012-13-2-r9)
Supplement: Additional file 1 — Supplemental tables and figures. [file gb-2012-13-2-r9-S1.PPT]

## Slide 1
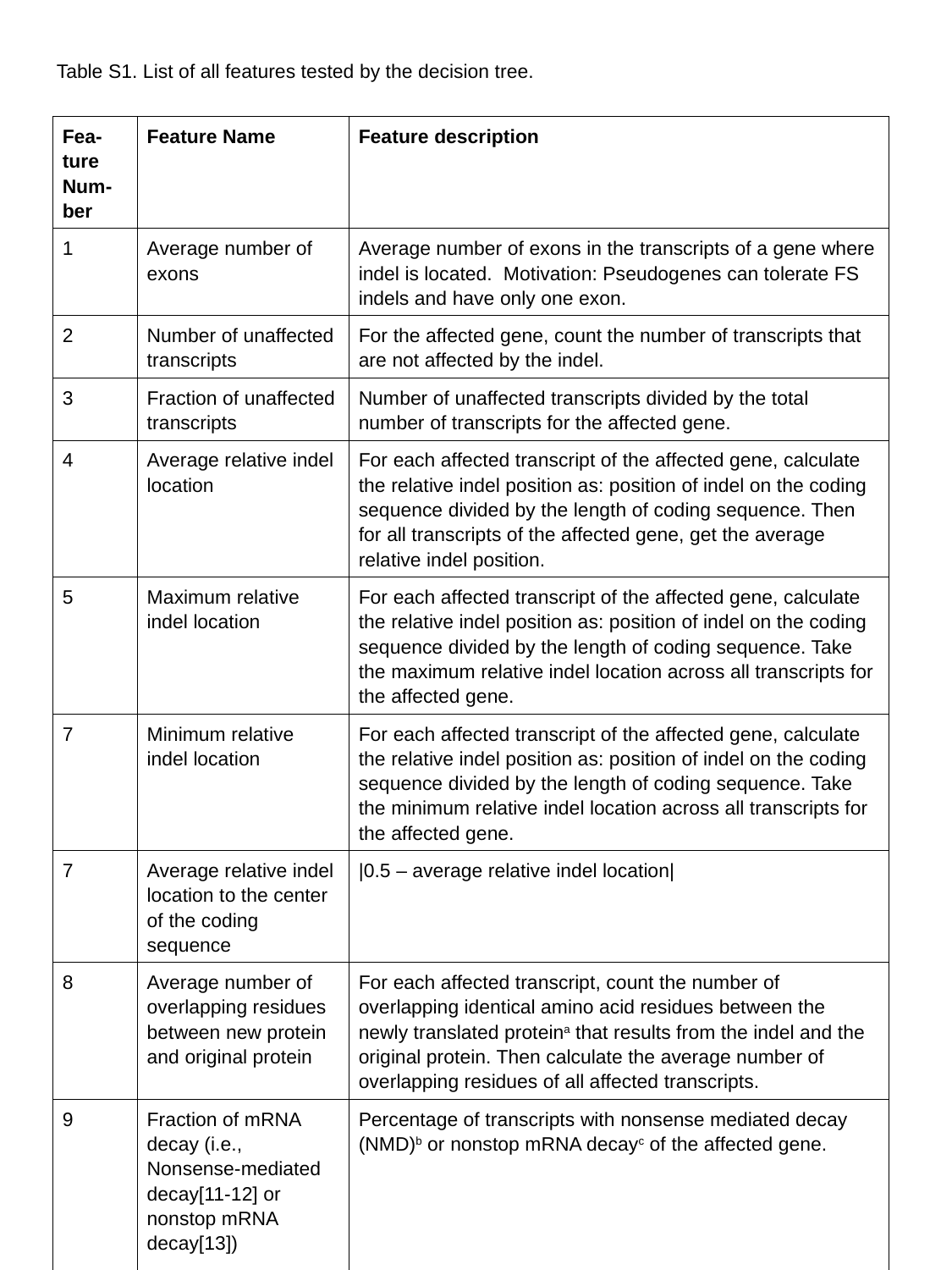

Table S1. List of all features tested by the decision tree.
| Fea- ture Num- ber | Feature Name | Feature description |
| --- | --- | --- |
| 1 | Average number of exons | Average number of exons in the transcripts of a gene where indel is located. Motivation: Pseudogenes can tolerate FS indels and have only one exon. |
| 2 | Number of unaffected transcripts | For the affected gene, count the number of transcripts that are not affected by the indel. |
| 3 | Fraction of unaffected transcripts | Number of unaffected transcripts divided by the total number of transcripts for the affected gene. |
| 4 | Average relative indel location | For each affected transcript of the affected gene, calculate the relative indel position as: position of indel on the coding sequence divided by the length of coding sequence. Then for all transcripts of the affected gene, get the average relative indel position. |
| 5 | Maximum relative indel location | For each affected transcript of the affected gene, calculate the relative indel position as: position of indel on the coding sequence divided by the length of coding sequence. Take the maximum relative indel location across all transcripts for the affected gene. |
| 7 | Minimum relative indel location | For each affected transcript of the affected gene, calculate the relative indel position as: position of indel on the coding sequence divided by the length of coding sequence. Take the minimum relative indel location across all transcripts for the affected gene. |
| 7 | Average relative indel location to the center of the coding sequence | |0.5 – average relative indel location| |
| 8 | Average number of overlapping residues between new protein and original protein | For each affected transcript, count the number of overlapping identical amino acid residues between the newly translated proteina that results from the indel and the original protein. Then calculate the average number of overlapping residues of all affected transcripts. |
| 9 | Fraction of mRNA decay (i.e., Nonsense-mediated decay[11-12] or nonstop mRNA decay[13]) | Percentage of transcripts with nonsense mediated decay (NMD)b or nonstop mRNA decayc of the affected gene. |

## Slide 2
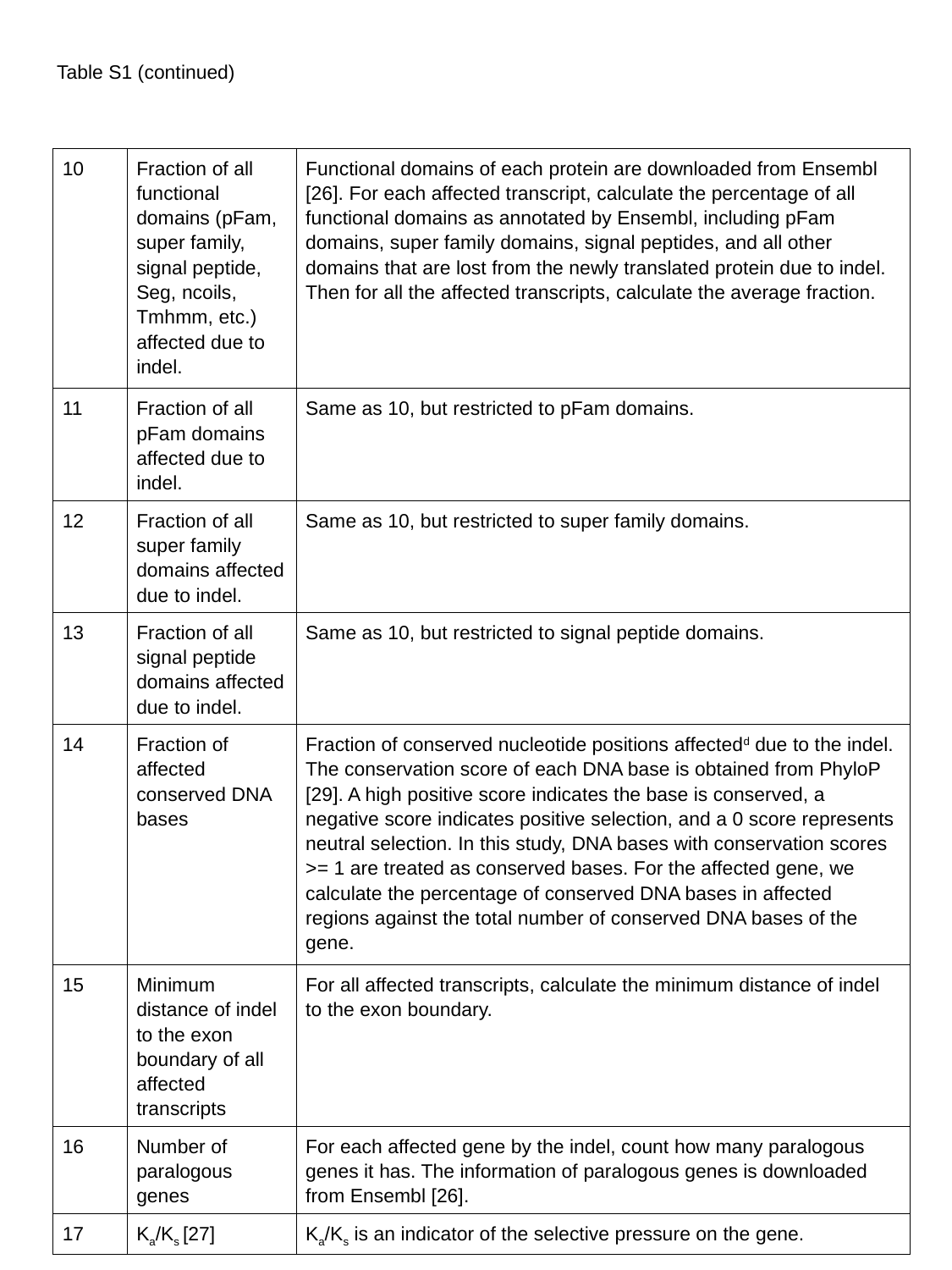

Table S1 (continued)
| 10 | Fraction of all functional domains (pFam, super family, signal peptide, Seg, ncoils, Tmhmm, etc.) affected due to indel. | Functional domains of each protein are downloaded from Ensembl [26]. For each affected transcript, calculate the percentage of all functional domains as annotated by Ensembl, including pFam domains, super family domains, signal peptides, and all other domains that are lost from the newly translated protein due to indel. Then for all the affected transcripts, calculate the average fraction. |
| --- | --- | --- |
| 11 | Fraction of all pFam domains affected due to indel. | Same as 10, but restricted to pFam domains. |
| 12 | Fraction of all super family domains affected due to indel. | Same as 10, but restricted to super family domains. |
| 13 | Fraction of all signal peptide domains affected due to indel. | Same as 10, but restricted to signal peptide domains. |
| 14 | Fraction of affected conserved DNA bases | Fraction of conserved nucleotide positions affectedd due to the indel. The conservation score of each DNA base is obtained from PhyloP [29]. A high positive score indicates the base is conserved, a negative score indicates positive selection, and a 0 score represents neutral selection. In this study, DNA bases with conservation scores >= 1 are treated as conserved bases. For the affected gene, we calculate the percentage of conserved DNA bases in affected regions against the total number of conserved DNA bases of the gene. |
| 15 | Minimum distance of indel to the exon boundary of all affected transcripts | For all affected transcripts, calculate the minimum distance of indel to the exon boundary. |
| 16 | Number of paralogous genes | For each affected gene by the indel, count how many paralogous genes it has. The information of paralogous genes is downloaded from Ensembl [26]. |
| 17 | Ka/Ks [27] | Ka/Ks is an indicator of the selective pressure on the gene. |

## Slide 3
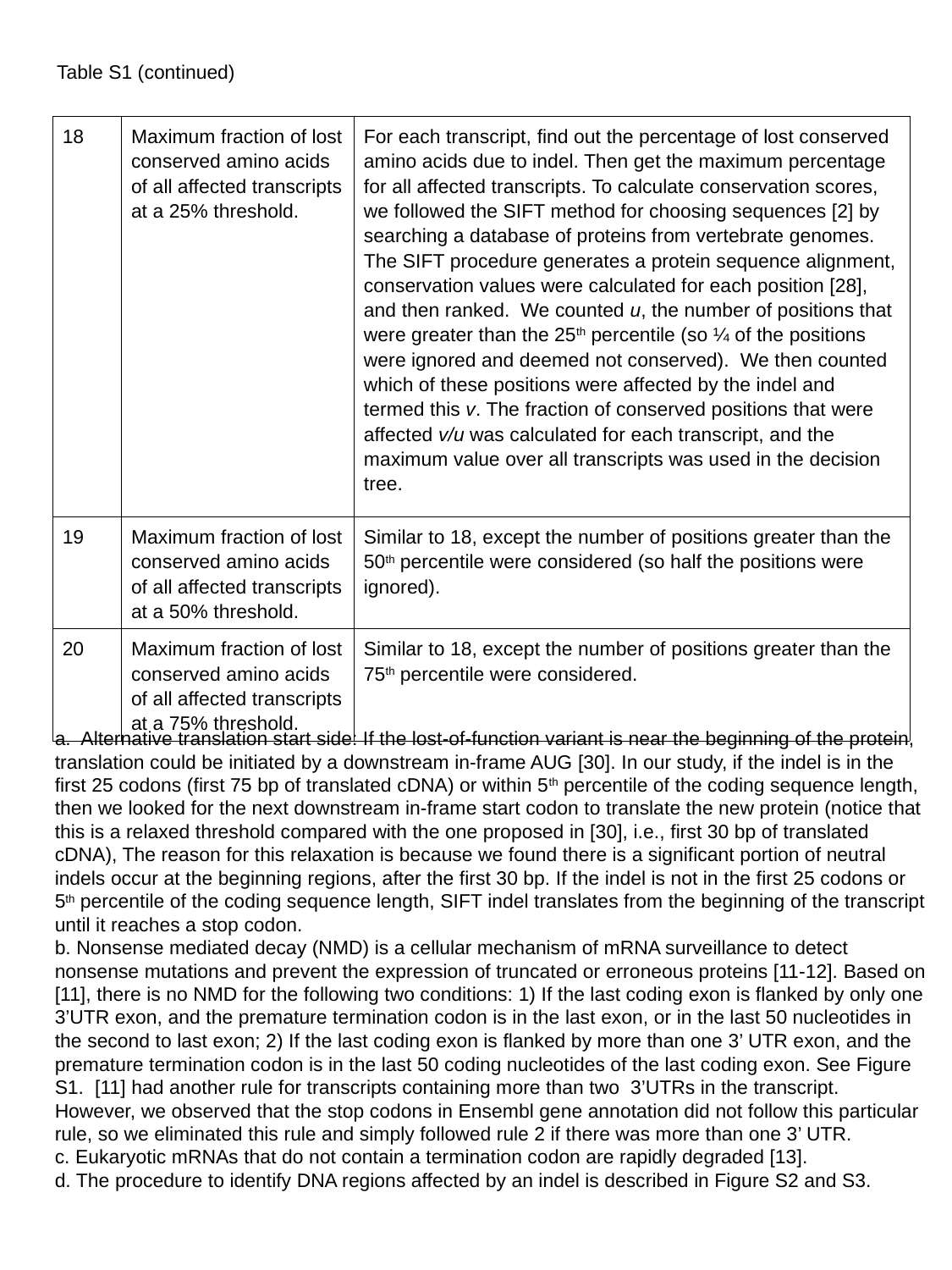

Table S1 (continued)
| 18 | Maximum fraction of lost conserved amino acids of all affected transcripts at a 25% threshold. | For each transcript, find out the percentage of lost conserved amino acids due to indel. Then get the maximum percentage for all affected transcripts. To calculate conservation scores, we followed the SIFT method for choosing sequences [2] by searching a database of proteins from vertebrate genomes. The SIFT procedure generates a protein sequence alignment, conservation values were calculated for each position [28], and then ranked. We counted u, the number of positions that were greater than the 25th percentile (so ¼ of the positions were ignored and deemed not conserved). We then counted which of these positions were affected by the indel and termed this v. The fraction of conserved positions that were affected v/u was calculated for each transcript, and the maximum value over all transcripts was used in the decision tree. |
| --- | --- | --- |
| 19 | Maximum fraction of lost conserved amino acids of all affected transcripts at a 50% threshold. | Similar to 18, except the number of positions greater than the 50th percentile were considered (so half the positions were ignored). |
| 20 | Maximum fraction of lost conserved amino acids of all affected transcripts at a 75% threshold. | Similar to 18, except the number of positions greater than the 75th percentile were considered. |
a. Alternative translation start side: If the lost-of-function variant is near the beginning of the protein, translation could be initiated by a downstream in-frame AUG [30]. In our study, if the indel is in the first 25 codons (first 75 bp of translated cDNA) or within 5th percentile of the coding sequence length, then we looked for the next downstream in-frame start codon to translate the new protein (notice that this is a relaxed threshold compared with the one proposed in [30], i.e., first 30 bp of translated cDNA), The reason for this relaxation is because we found there is a significant portion of neutral indels occur at the beginning regions, after the first 30 bp. If the indel is not in the first 25 codons or 5th percentile of the coding sequence length, SIFT indel translates from the beginning of the transcript until it reaches a stop codon.
b. Nonsense mediated decay (NMD) is a cellular mechanism of mRNA surveillance to detect nonsense mutations and prevent the expression of truncated or erroneous proteins [11-12]. Based on [11], there is no NMD for the following two conditions: 1) If the last coding exon is flanked by only one 3’UTR exon, and the premature termination codon is in the last exon, or in the last 50 nucleotides in the second to last exon; 2) If the last coding exon is flanked by more than one 3’ UTR exon, and the premature termination codon is in the last 50 coding nucleotides of the last coding exon. See Figure S1. [11] had another rule for transcripts containing more than two 3’UTRs in the transcript. However, we observed that the stop codons in Ensembl gene annotation did not follow this particular rule, so we eliminated this rule and simply followed rule 2 if there was more than one 3’ UTR.
c. Eukaryotic mRNAs that do not contain a termination codon are rapidly degraded [13].
d. The procedure to identify DNA regions affected by an indel is described in Figure S2 and S3.

## Slide 4
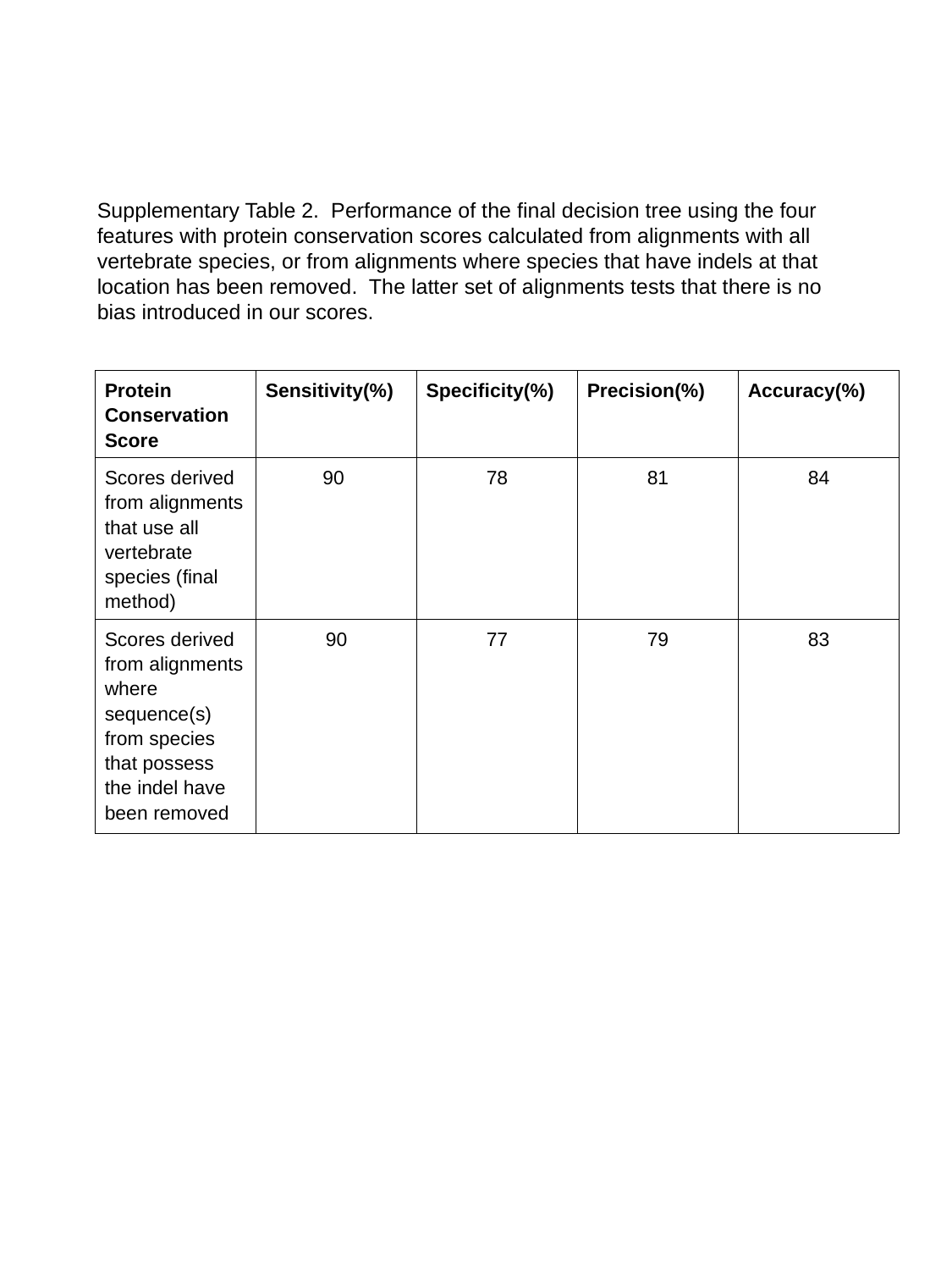

Supplementary Table 2. Performance of the final decision tree using the four features with protein conservation scores calculated from alignments with all vertebrate species, or from alignments where species that have indels at that location has been removed. The latter set of alignments tests that there is no bias introduced in our scores.
| Protein Conservation Score | Sensitivity(%) | Specificity(%) | Precision(%) | Accuracy(%) |
| --- | --- | --- | --- | --- |
| Scores derived from alignments that use all vertebrate species (final method) | 90 | 78 | 81 | 84 |
| Scores derived from alignments where sequence(s) from species that possess the indel have been removed | 90 | 77 | 79 | 83 |

## Slide 5
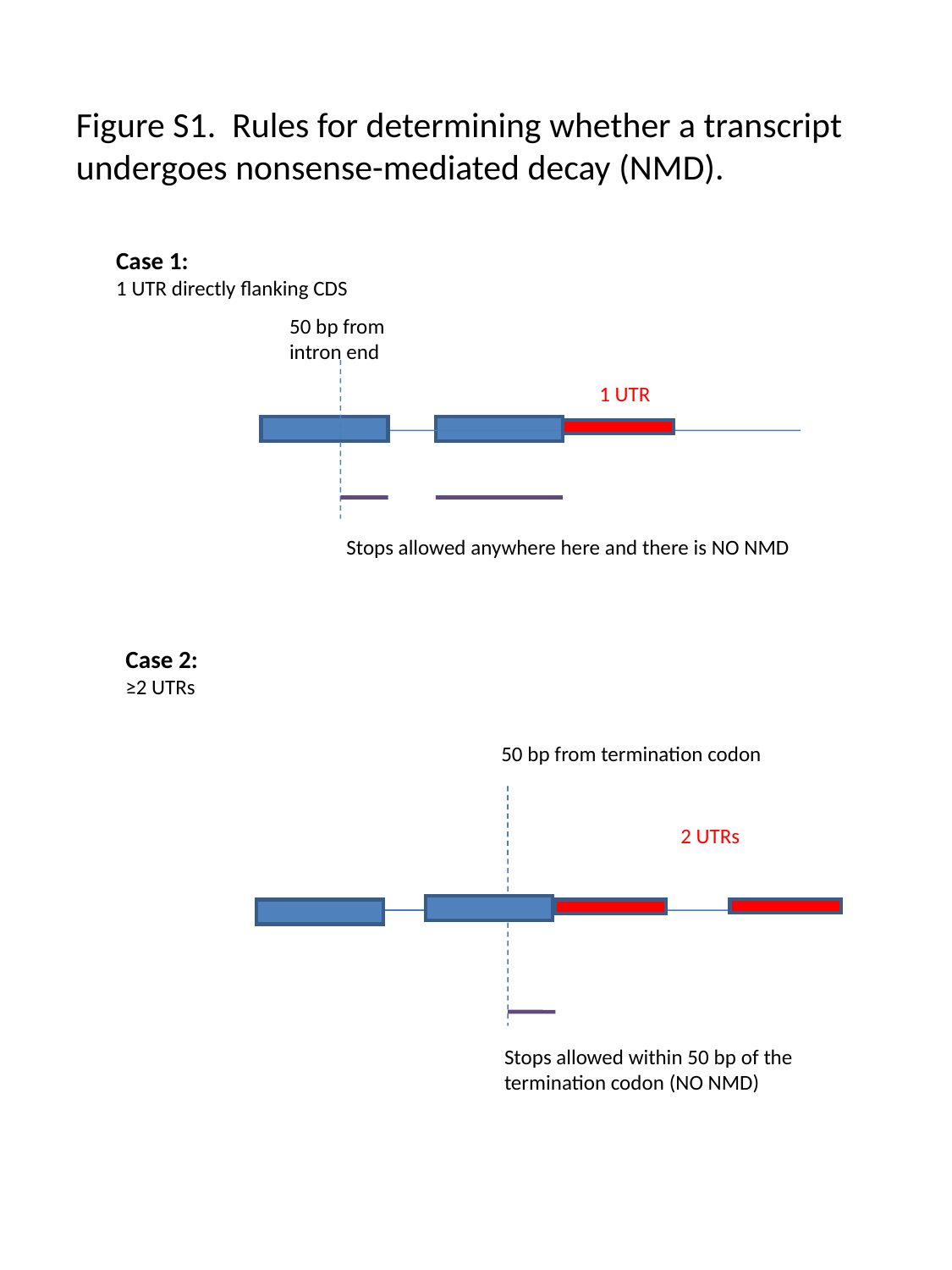

# Figure S1. Rules for determining whether a transcript undergoes nonsense-mediated decay (NMD).
Case 1:
1 UTR directly flanking CDS
50 bp from intron end
1 UTR
Stops allowed anywhere here and there is NO NMD
Case 2:
≥2 UTRs
50 bp from termination codon
2 UTRs
Stops allowed within 50 bp of the termination codon (NO NMD)

## Slide 6
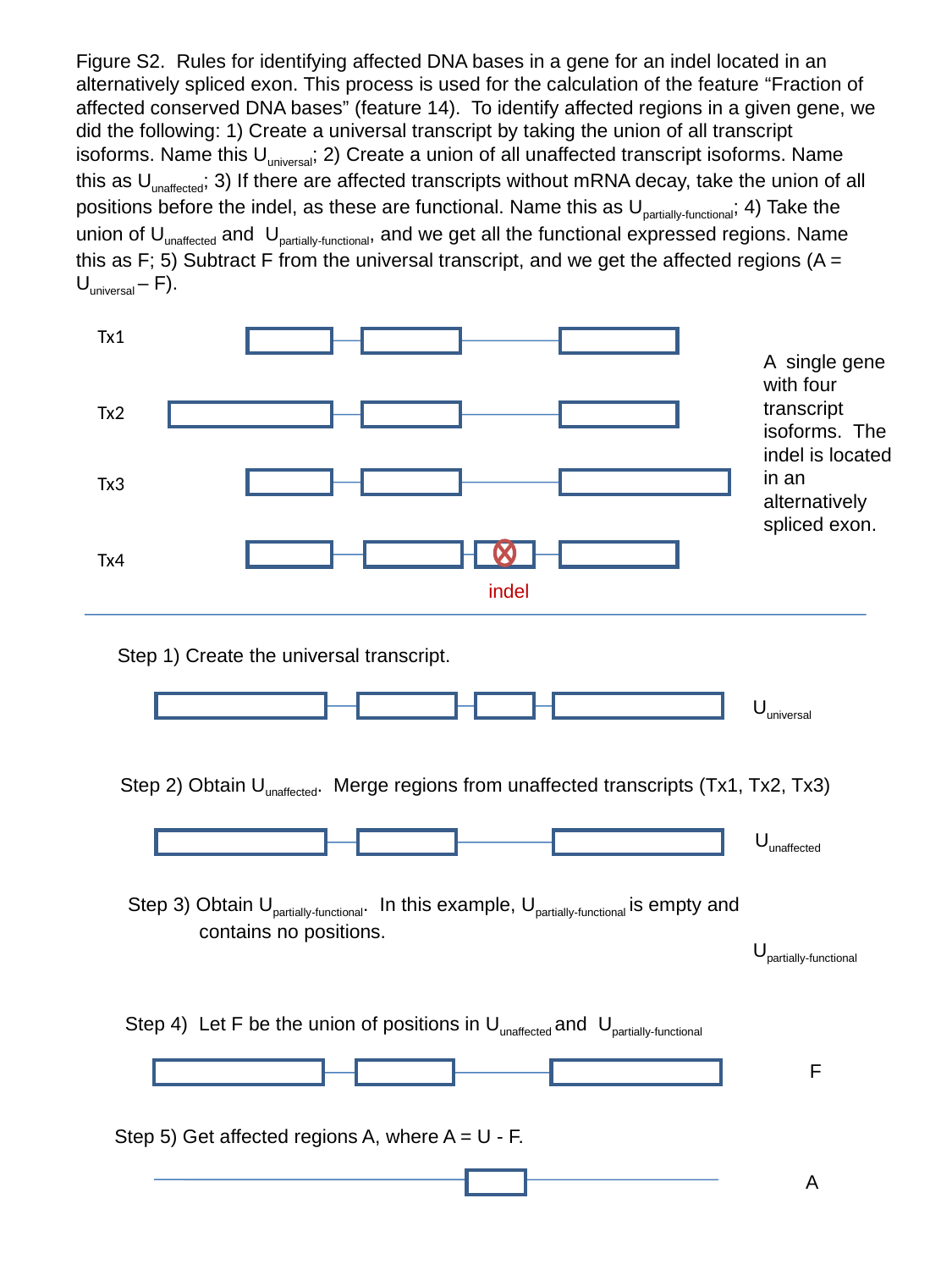

Figure S2. Rules for identifying affected DNA bases in a gene for an indel located in an alternatively spliced exon. This process is used for the calculation of the feature “Fraction of affected conserved DNA bases” (feature 14). To identify affected regions in a given gene, we did the following: 1) Create a universal transcript by taking the union of all transcript isoforms. Name this Uuniversal; 2) Create a union of all unaffected transcript isoforms. Name this as Uunaffected; 3) If there are affected transcripts without mRNA decay, take the union of all positions before the indel, as these are functional. Name this as Upartially-functional; 4) Take the union of Uunaffected and Upartially-functional, and we get all the functional expressed regions. Name this as F; 5) Subtract F from the universal transcript, and we get the affected regions (A = Uuniversal – F).
Tx1
A single gene with four transcript isoforms. The indel is located in an alternatively spliced exon.
Tx2
Tx3
Tx4
indel
Step 1) Create the universal transcript.
Uuniversal
Step 2) Obtain Uunaffected. Merge regions from unaffected transcripts (Tx1, Tx2, Tx3)
Uunaffected
Step 3) Obtain Upartially-functional. In this example, Upartially-functional is empty and
 contains no positions.
Upartially-functional
Step 4) Let F be the union of positions in Uunaffected and Upartially-functional
F
Step 5) Get affected regions A, where A = U - F.
A

## Slide 7
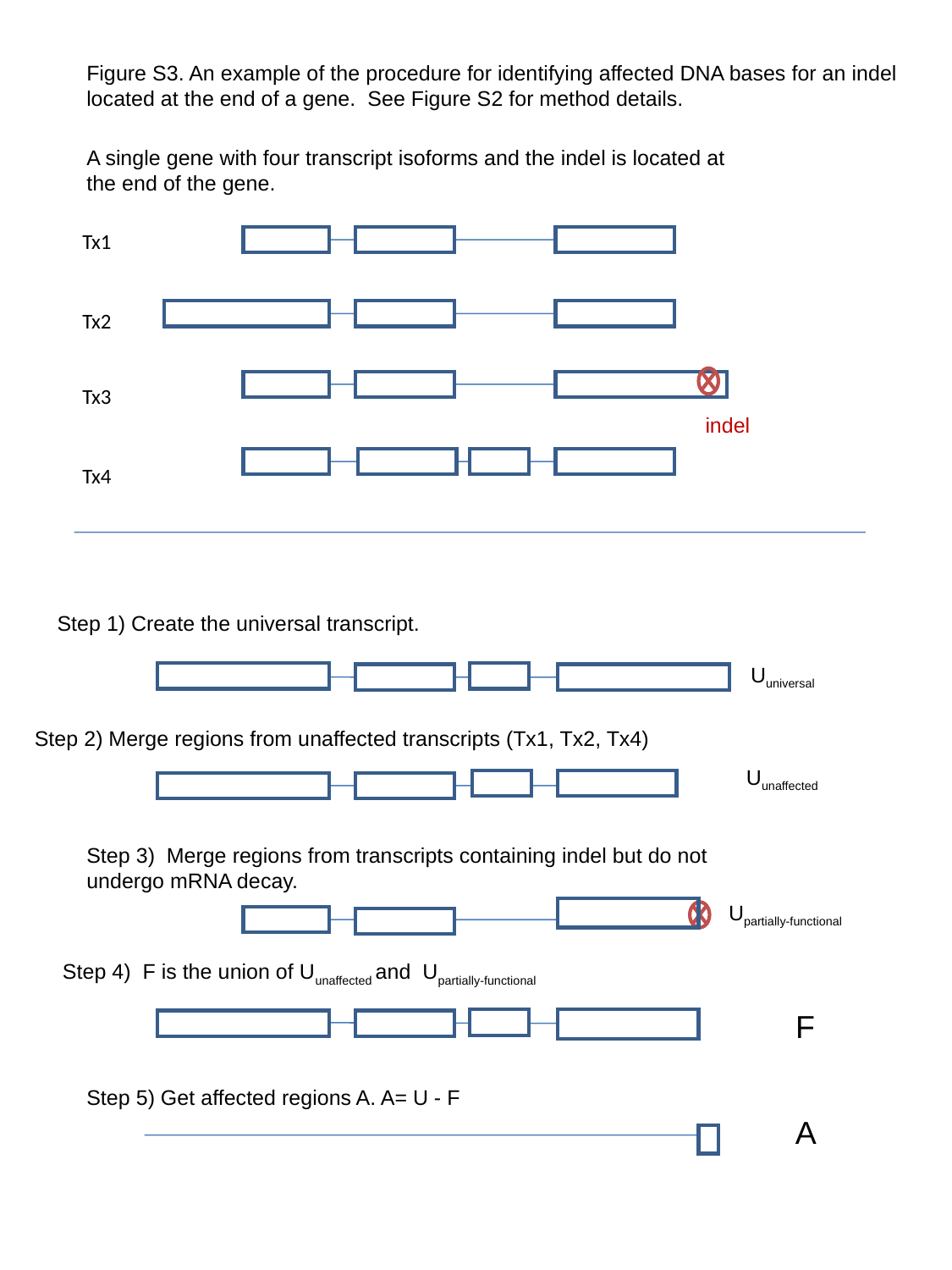

Figure S3. An example of the procedure for identifying affected DNA bases for an indel located at the end of a gene. See Figure S2 for method details.
A single gene with four transcript isoforms and the indel is located at the end of the gene.
Tx1
Tx2
Tx3
indel
Tx4
Step 1) Create the universal transcript.
Uuniversal
Step 2) Merge regions from unaffected transcripts (Tx1, Tx2, Tx4)
Uunaffected
Step 3) Merge regions from transcripts containing indel but do not undergo mRNA decay.
Upartially-functional
Step 4) F is the union of Uunaffected and Upartially-functional
F
Step 5) Get affected regions A. A= U - F
A
